# Review of clinical practice utility of positron emission tomography with 18F-fluorodeoxyglucose in assessing tumour response to therapy

**DOI:** 10.1007/s11547-014-0446-4

**Published:** 2014-08-26

**Authors:** Andrea d’Amico

**Affiliations:** Department of Diagnostic Positron Emission Tomography, Maria Skłodowska-Curie Memorial Cancer Centre and Institute of Oncology, ulica Wybrzeże Armii Krajowej 15, 44-101 Gliwice, Poland

**Keywords:** Cancer, Treatment, Imaging, Metabolism

## Abstract

Positron emission tomography, most commonly with 18F-fluorodeoxyglucose, is being used for evaluation of tumour response to therapy. Limitations of this method are associated with (1) fluorodeoxyglucose pharmacokinetic properties, (2) the detection system, (3) discrepancies between metabolic and anatomic images, and (4) acquisition standardization. Response to therapy may be evaluated with qualitative (Deauville score), semiquantitative (standardised uptake value), and quantitative methods (European Organization for Research and Treatment of Cancer; Positron Emission Tomography Response Criteria in Solid Tumours). Methods under evaluation include metabolic tumour volume, total lesion glycolysis, and heterogeneity of fluorodeoxyglucose uptake. The development of positron emission tomography scanners that have larger fields of view may facilitate tumour assessment based on kinetic modelling. Increased clinical use of these methods will depend on the development and validation of intuitive and simple analytic tools.

## Introduction

Most malignancies incorporate glucose much more than normal tissues. The glucose analogue fluorodeoxyglucose (FDG) labelled with the positron emitter fluorine 18 is the basis of positron emission tomography (PET) scanning in oncological imaging. Glucose and FDG enter the cell via the membrane glucose transporter, and phosphorylation at position number six prevents glucose and FDG from escaping the cell. Unlike glucose, FDG cannot be catabolised in the glycolytic pathway and remains in the form FDG-6-phosphate for the duration that the molecule remains radioactive and visible on PET. The distribution of radiolabelled FDG reflects the biodistribution of glucose and its phosphorylation in the different regions of the body.

In 1931, the German scientist Otto Heinrich Warburg showed a relation between the degree of conversion of glucose to lactic acid and tumour growth, and this became the basis of cancer imaging with FDG. The Warburg effect is a complex of biochemical alterations in the neoplastic cell that includes markedly increased aerobic glycolysis in the cytosol and impaired mitochondrial oxidative phosphorylation. The production of adenosine triphosphate by the metabolism of glucose to lactate is caused by up-regulation of several enzymes and transporters (glucose transporter 1, hexokinase II, pyruvate kinase M2, and lactate dehydrogenase) [1, 2]. Despite the lower efficiency of energy production by aerobic glycolysis than mitochondrial respiration, glucose catabolism may generate precursors for the synthesis of proteins, nucleic acids, and membranes that are essential for cell proliferation. Therefore, the Warburg effect occurs in cancers and other rapidly proliferating eukaryotic cells such as yeast cultures and is a universal mechanism that may provide growth advantages [3–5].

A small amount of FDG, much lower than obtained with pharmacological doses, may cause a concentration of radioactivity within a tumour that may be detected easily with modern PET computed tomography (PET-CT) or PET magnetic resonance imaging (PET-MRI) devices. The degree of FDG accumulation in healthy tissues may enable the identification of pathological areas in most body regions, with some restriction for organs that have exclusively glucose-based metabolism such as the brain or that participates in FDG excretion such as the kidneys and urinary system.

The most common applications of FDG-PET in oncology involve the evaluation of disease extent. For tumours that have high accumulation of FDG, most tumours that exceed the resolution of the acquisition system are visible on PET images except for artefacts. This fact is very important for lymph node localisation, and diagnostic accuracy is greater for FDG-PET scanning than CT or MRI scanning without PET [6, 7].

False negative results with PET scanning may occur with tumour pathologies that have low levels of FDG accumulation such as prostate, stomach, or neuroendocrine tumours. False negative results also may occur with lesions that are below the limits of resolution of the PET scanner (currently, 4–5 mm) or lesions that are anatomically close to a structure that is moving during the PET examination such as the diaphragm. However, false negative results may be minimised with careful patient selection and awareness of the inherent limitations of the method.

False positive results are currently a greater clinical problem than false negative results because many non-neoplastic diseases may have a high level of glucose metabolism, such as infections or inflammatory conditions with lymph node involvement. In addition, physiological activation of muscles, bowels, brown fat, and other normal tissues may cause misleading findings on PET scans. However, false positive results may be minimised with careful patient preparation and experience of the nuclear medicine specialist.

Imaging with FDG-PET is useful because of the early and marked reduction in tumour metabolism in response to chemotherapy or radiation therapy. The evaluation of the response to therapy involves the comparison of tests before and after treatment. The FDG-PET scan may detect a treatment response in a very early phase of treatment, and this may enable the identification of chemoresistant tumours that may benefit from alternative treatments. In addition, it may be important to detect residual disease after treatment is completed.

Current PET scans are usually performed with PET-CT scanning, but PET-MRI scanners are becoming available. The clinical value of PET-CT is well established, but the benefits of PET-MRI are being evaluated. The ability to detect the photons emitted from the patient is similar, and the quality of metabolic imaging is comparable, between PET-CT and PET-MRI devices. These methods differ primarily in the morphological method of imaging. The PET-MRI scan may provide greater inherent contrast to visualise soft tissues, which could be advantageous in evaluating pelvic tumours and sarcomas, but PET-CT is better for oncological imaging of the lungs. In children, PET-MRI is preferred because of the lower radiation dose than with PET-CT scanning [8].

## Quantification of tumour with positron emission tomography

Nuclear medicine techniques including PET scanning have limitations that differ from other radiographic studies. These limitations are associated with (1) FDG pharmacokinetic properties, (2) the detection system, (3) discrepancies between metabolic and anatomic images, and (4) acquisition standardization.

The pharmacokinetic properties of FDG enable a dynamic method of image acquisition. The most important mathematical model to analyse the dynamic PET data is compartmental analysis. The sequential data obtained after administration of the radiotracer, (corrected for attenuation, scatter, and radioactive decay) enable determination of the concentration of radiotracer in the body region. To interpret the PET data, it is assumed that the measured radiotracer can be considered in physiologically distinct compartments. The most common compartmental model for the analysis of PET data is the Patlak plot, a graphical analysis technique that assumes three compartments: the arterial blood compartment, the compartment of radiotracer-free distribution, and the compartment of specific and irreversible binding [9]. Quantitative assessment of tumour metabolism is performed with pharmacokinetic constants that express radiotracer passage between compartments.

Clinical evaluation of dynamic data may be limited because of the need to shorten the time of image acquisition for each patient, the difficulty of arterial blood sampling to evaluate the input function, and the small field of view of the PET scanner (approximately 20 cm). In addition, small differences in time between FDG administration and PET scanning for different scans (before and after treatment) may cause major errors in interpreting radiotracer accumulation.

Current PET scanners have a spatial resolution of approximately 4 mm. Radiotracer uptake in lesions that have diameter <3 times the scanner resolution is affected by partial volume effect, which causes loss of apparent activity. For example, in a PET scanner that has spatial resolution 4 mm, the image of a radioactive phantom with 6-mm diameter may show a maximum measured 60 % real activity concentration [10].

Modern PET-CT devices typically require ≥10 min between the initial CT scan and PET image acquisition in more distal body parts. Therefore, misalignment is usually present between metabolic and anatomical images. Furthermore, the acquisition of each PET image requires ≥90 s, and breathing movements make it impossible to quantify accurately the metabolism of lesions near the diaphragm. Acquisition techniques such as PET gating for breathing may be helpful, but these methods lengthen the time of acquisition and data processing and are not used frequently in clinical settings [11].

Standardised acquisition protocols of the European Society for Therapeutic Radiology and Oncology and European Association of Nuclear Medicine include information about the calibration of the PET scanner, radiotracer uptake time, and approach for definition of regions of interests. These protocols are used to derive quantitative parameters from calculated values such as standardised uptake value (SUV) [12]. In contrast with CT scanning, it is not possible to compare quantitative measurements of PET scans performed in different institutions.

## Qualitative and visual evaluation methods

Comparative visual assessment of PET images is commonly practised and frequently enables reliable judgements about decreased tumour metabolism after therapy. The correct reconstruction of PET images should be checked in advance. The projected data are detected on every pair of photons originated from positron annihilation forms. A sinogram is a graphic expression of the raw data that enables detection of abnormalities and verification of consistency between pretreatment and control PET scans [13]. However, qualitative assessment is less useful when a tumour has partial reduction of metabolism after treatment and incomplete normalisation. In these cases, the uptake by the tumour may be similar to physiological activity present in other regions that have a stable level of metabolism. Reference organs typically include the liver and mediastinal tissues.

Early investigation of malignancy by PET was performed with lymphoma, and the earliest systems to assess treatment were proposed for these neoplasms. A variable portion of a lymphoma mass consists of malignant cells, and many patients have residual masses after completion of therapy. Morphological examination is insufficient to exclude the presence of disease in residual masses or early recurrence in normal sized lymph nodes. In addition, the shrinkage of the tumour may occur late after treatment and is a not a proper criterion in early evaluation. In contrast, the metabolic response may be detected after 1–3 cycles of chemotherapy and is correlated with overall and disease-free survival [14–17].

The Deauville score is a popular method for the evaluation of qualitative response to treatment of Hodgkin lymphoma. This score is based on a simple classification of the degree of pathological FDG uptake at the tumour compared with the liver and mediastinum (Table 1) [18]. Lesions that have FDG radiotracer uptake ≤ liver (Deauville score 1, 2, or 3) are metabolically negative, and lesions that have FDG uptake > liver (Deauville score 4 or 5) are positive (Table 1) [18]. The Deauville score was validated in a multicentre trial with many patients [19].

**Table 1:** Deauville scoring system for evaluation of Hodgkin lymphoma with ^18^F-fluorodeoxyglucose positron emission tomography

Deauville Score	Radiotracer uptake
1	No lesion uptake > background
2	Lesion ≤ mediastinum
3	Mediastinum < lesion ≤ liver
4	Lesion moderately > liver
5	Lesion markedly > liver
x	New lesion uptake unlikely related to lymphoma

Adapted from Meignan et al. [11]

In contrast, PET scanning is unsuitable for assessing the activity and extent of tumours that have moderate radiotracer accumulation, such as renal clear cell carcinoma, primary liver tumours, and adenocarcinoma of the prostate [20].

## Methods based on the standardised uptake value

**Fig. 1:**
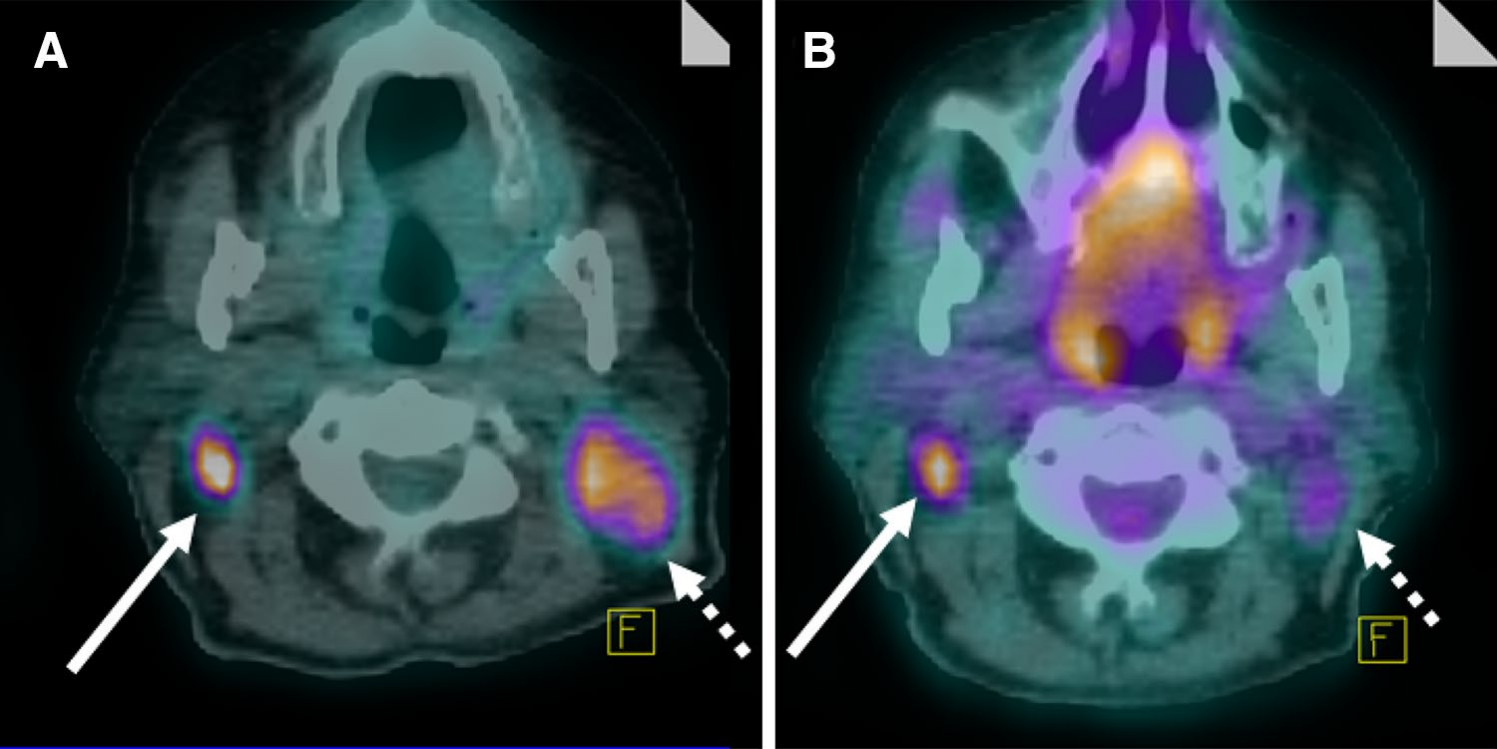
Fluorodeoxyglucose positron emission tomography-computed tomography (FDG PET-CT) images. This patient had lymph node metastatases from a planocellular nasopharyngeal carcinoma of the right neck (*solid arrow*) coexisting with Hodgkin lymphoma at the left neck (*dotted arrow*). **a** Pretreatment FDG PET-CT scan showed pathological FDG uptake at the carcinoma and lymphoma. **b** The FDG PET-CT scan after two cycles of chemotherapy (cisplatin, doxorubicin, and cyclophosphamide) showed a partial metabolic response of carcinomatous localisation (maximum standardised uptake value [SUVmax], −54 %; total lesion glycolysis, −47 %) and complete metabolic regression of lymphomatous localisation (SUVmax, −69 %; total lesion glycolysis, −82 %; downshifted from Deauville 4 to Deauville 2)

The SUV, a semiquantitative indicator of FDG uptake by tumours, is the ratio between the concentration of radioactivity measured in a body part and the hypothetical concentration of radioactivity that should be measured with a homogeneous distribution of radiotracer in the entire body [21]. Activity in a tumour may be expressed as the voxel that has maximum SUV in the whole tumour volume (SUVmax):

$$ {\text{SUV}}\hbox{max} = [C\left( {\mu {\text{Ci}}/{\text{mL}}} \right)/{\text{ID}}\left( {\mu {\text{Ci}}} \right)]/w , $$

where *C* is the activity at a pixel within the tissue identified by regions of interest, and ID is the injected dose per kilogram of the patient’s body weight (*w*). The SUVmax may be measured quickly and easily and is affected less by the partial volume effect than mean tumour SUV (SUVmean) (Fig. 1).

When the SUVmax is used to monitor a tumour, the change in activity (ΔSUV) from initial (SUVmax1) to later scans (SUVmax2) may be calculated as:

$$ \Delta {\text{SUV}}\left( \% \right) = 100 \times \left( {\left[ {{\text{SUV}}\hbox{max} 2/{\text{SUV}}\hbox{max} 1} \right] - 1} \right). $$

In addition, the SUV is normalised to a reference activity, typically the FDG uptake by the liver.

The reliability of SUVmax measurement is affected by many factors including attenuation correction, scatter correction, respiratory motion, partial volume effect, and tomographic reconstruction. The problem of reliability necessitates highly standardised protocols for PET imaging to provide the same conditions of acquisition and data processing between PET examinations for proper comparisons [22].

The first quantitative criteria to monitor tumours were proposed by the European Organization for Research and Treatment of Cancer (EORTC) in 1999. The EORTC criteria defined tumour response to treatment as changes in SUV > 15 % after the first cycle of chemotherapy; changes in SUV > 25 % afte >1 cycle of chemotherapy; changes in FDG accumulation >20 %; or new foci of pathological FDG uptake [23].

The PET response criteria in solid tumours (PERCIST) is a more detailed classification and requires a more rigorous standardisation for data acquisition and analysis than the EORTC criteria (Table 2) [24]. The PERCIST criteria define limits for maximum acceptable glycaemia, injected dose, and acquisition timing, and these criteria are suitable only for examinations performed with hybrid PET-CT scanners. The tumour activity is quantified by the SUV peak, which considers the average concentration of radioactivity in a spherical area (radius, 1 cm) centred on the most active part of the tumour. Differences in SUV peak >30 % are important. The PERCIST criteria may be applied only to lesions >2 cm to avoid errors caused by the partial volume effect. In addition, an analysis of the reference uptake (hepatic or mediastinal) is required in PERCIST to ensure the absence of significant differences in FDG biodistribution from before to after therapy. Commercial programs for the application of PERCIST are available for most PET workstations.

**Table 2:** Classification systems for positron emission tomography evaluation of response to treatment of solid tumours, adapted from Wahl et al. [15]

Parameter	EORTC	PERCIST
Lesion measure	SUVmax and SUVmean in a manually drawn region of interest	SUV peak of the tumour lesion with greatest uptake (primary or metastatic)
Reproducibility	±25 % liver uptake	±20 % and <0.3 SUL on liver
Timing	ND	<15 min difference from injection to acquisition between scans before and after treatment
Acquisition and calibration	ND	Same scanner and reconstruction software should be used for scans before and after treatment Proper calibration required
Complete metabolic response (CMR)	No pathological FDG uptake foci	No FDG uptake foci below mean liver activity No new foci
Partial metabolic response (PMR)	After first chemotherapy cycle: SUV reduction 15–25 % After subsequent chemotherapy cycles: SUV reduction >25 %	SUL reduction >30 % in target lesion with minimum 0.8 SUL decrease No increase in SUL or size in non-target lesions
Progressive metabolic disease (PMD)	Increase >25 % tumour SUV or increase >20 % tumour longest dimension or appearance of new lesions	SUL increase >30 % in target lesion (minimum, 0.8 SUL) or visible increase of lesion extent (minimum >75 % total lesion glycolysis) or appearance of new lesions
Stable metabolic disease (SMD)	Increase <25 % or decrease <15 % tumour SUV	Not CMR, PMR, or PMD

*CMR* complete metabolic response, *EORTC* European Organization for Research and Treatment of Cancer, *FDG* fluorodeoxyglucose, *ND* not defined, *PERCIST* positron emission tomography response criteria in solid tumours, *PMD* progressive metabolic disease, *PMR* partial metabolic response, *SMD* stable metabolic disease, *SUL* standardised uptake value for lean body mass, *SUV* standardised uptake value

Several systems have been proposed to correct for the partial volume effect. These techniques are based on image deconvolution, model-based reconstructions, or the simultaneous assessment of radiographic CT or MR images that are properly coregistered with PET. No system completely eliminates the partial volume effect, but some are valid for improving the precision of SUV measurement in small tumours. These models still are being validated and were not considered in the definition of the EORTC and PERCIST criteria. However, it is important to quantify tumours <2 cm correctly, and partial volume correction methods are being developed [25–28].

## Methods under evaluation

### Metabolic tumour volume

Several methodological errors may confound the quantification of tumour metabolism when using the SUV parameter. Therefore, other metabolic indices have been proposed that consider the radiotracer uptake in the entire tumour mass. Radiographic morphological methods cannot quantify the number of malignant cells because there is structural inhomogeneity in neoplastic tissue. However, the uptake of FDG is indicative of the presence of viable tumour cells. Therefore, PET may be used to estimate the volume of biologically active tumour (metabolic tumour volume).

The main problem in quantifying metabolic tumour volume is the definition of the threshold SUV between the viable tumour and background tissue. Tumour contours may be defined manually, but manual segmentation is time consuming and lacks repeatability. Alternatively, the SUV threshold may be calculated as the percent maximal tumour uptake of FDG in tissues surrounding the tumour or other reference organs such as the liver [29–32]. In addition, other systems are available that are not based on the SUV threshold and that use complex mathematical models to detect the tumour-background interface [33–39]. Very high repeatability is feasible with some techniques [4].

### Total lesion glycolysis

The calculation of metabolic tumour volume is based on the definition of tumour boundaries. Therefore, metabolic tumour volume does not indicate possible differences in the density of neoplastic cells within the tumour. The total lesion glycolysis is the product of the average tumour SUV (which is an index of the density of neoplastic cells) and metabolic tumour volume. The total lesion glycolysis may correlate with overall survival in patients who have lung cancer, colon cancer with liver metastasis, and non-Hodgkin lymphoma (Fig. 1) [40–43].

### Heterogeneity of fluorodeoxyglucose uptake

In addition to the volumetric parameters, additional features may be extracted from PET images by computational methods such as tumour texture and heterogeneity [44]. The heterogeneity of FDG distribution within the tumour mass may be a useful index of response to treatment. Tumour progression is associated with cell proliferation, hypoxia, and necrosis, which may decrease homogeneity of FDG distribution in the tumour. Mathematical models that measure the level of heterogeneity may be useful in selected patients [45, 46].

## Conclusions

It is feasible to perform early measurement of the effect of therapy on cancer, and functional tomographic techniques such as FDG-PET may be useful in addition to morphological imaging methods. The accuracy in measuring tumour response is limited by the difficulty in performing dynamic analysis of tumour metabolism after the radiopharmaceutical agent is given. Tumour activity may be affected by many variables about the patient and conditions of data acquisition. The development of PET scanners that have larger fields of view may facilitate tumour assessment based on kinetic modelling. In addition, signal degradation because of the partial volume effect may limit the evaluation of small lesions. Measurement error may be decreased by validating correction algorithms and integrating these algorithms with response criteria such as EORTC and PERCIST. Models that are based on total lesion glycolysis or tumour heterogeneity may be useful in selected patients. Increased clinical use of these methods will depend on the development and validation of intuitive and simple analytic tools.
